# Sampling Efficacy and Survival Rates of *Labarrus pseudolividus* (Coleoptera: Scarabaeidae) and *Onthophagus taurus* (Coleoptera: Scarabaeidae) Using Flotation and Sieve-Separation Methodology

**DOI:** 10.1093/jisesa/ieaa083

**Published:** 2020-11-02

**Authors:** Fallon Fowler, Tashiana Wilcox, Stephanie Orr, Wes Watson

**Affiliations:** Department of Entomology and Plant Pathology, North Carolina State University, Grinnells Animal Health Laboratories, Raleigh, NC

**Keywords:** Scarabaeidae, survey, detection, monitoring, ecology

## Abstract

Understanding collection methodologies and their limitations are essential when targeting specific arthropods for use in habitat restoration, conservation, laboratory colony formation, or when holistically representing local populations using ecological surveys. For dung beetles, the most popular collection methodology is baited traps, followed by light traps and unbaited flight-intercept traps during diversity surveys. A less common collection method, flotation, is assumed to be laborious and messy, and so only a handful of papers exist on its refinement and strengths. Our purpose was threefold: First, we tested the recovery and survival rates of *Labarrus* (=*Aphodius*) *pseudolividus* (Balthasar) and *Onthophagus taurus* (Schreber) when floating beetle-seeded dung pats to determine potential collection and safety issues. We collected 72.4 and 78% of the seeded *L. pseudolividus* and *O. taurus*, respectively, with >95% survival rating. Second, we developed a flotation-sieving technique that enables users to rapidly collect and passively sort dung beetles with less time and effort. Specifically, we often collected 50–100 g of wild dung beetles within a couple of hours of gathering dung and sorted them in a couple more by allowing dung beetles to sort themselves by size within a series of sieves; Third, we reviewed flotation-based advantages and disadvantages in comparison to other methodologies.

Dung beetles improve resource cycling, pasture health, and animal welfare primarily by: reducing pest, parasite, and disease incidence and enhancing soil fertility through humification, refilling water tables, and reducing point-source fecal pollution (Nichols et al. 2008, Doube 2018). Dung beetles are important ecosystem engineers and are representative bioindicators of localized diversity, as they rely mostly on animal dung for consumption and reproduction (Davis and Scholtz 2001). However, many ‘dung’ beetles may consume non-dung materials such as carrion (vertebrate and invertebrate) and other rotting material (fruit, fungus, compost) (Flechtmann et al. 2009, De Andrade et al. 2011). Therefore, effectively and efficiently trapping dung beetles on a time and monetary budget is essential for ecological surveys, introducing populations for ecological restoration (Doube 2018), and for gathering information on their biological utility.

Dung beetle collection methods are numerous, with each possessing their own unique strengths and weaknesses, these include unbaited flight-intercept traps, baited pitfalls or hanging traps, light traps, and resource-based extraction via flotation, light, or sieving. Of these, the unbaited flight-intercept trap is rarely used as it randomly targets all flying insects rather than dung beetles *per se*; meanwhile, baited, light, and extraction traps offer targeted, time-specific snapshots of local dung beetle communities. In particular, baited and light-based methods are excellent for species-specific collection or local monitoring because they capture the most attracted and mobile beetles drawn to the trap with minimal effort (De Andrade et al. 2011)—this is particularly useful in areas where colonized substrates are rare or patchy. In contrast, extraction-based methods survey species currently occupying the natural resource and provide a substrate-based snapshot (Krell 2007); thus, this methodology performs best when dung beetles are aggregated within dung pats (thereby reducing the dung amount collected) and where dung easily found (e.g., along water or shade sources). Both baited and light-based methodologies select dung beetles based on their daily activity patterns (e.g., diurnal, crepuscular, nocturnal) or attractiveness. Baited trap effectiveness—often correlated with bait attractiveness—is affected by the size, type, moisture content, and age of dung, or even other accidental additives, such as accumulated rotting invertebrates, which can select or repel specific dung beetle groups (Peck and Howden 1984, Finn and Giller 2000, Flechtmann et al. 2009). Light traps, similarly, can skew dung beetle representation by attracting dung beetles with preferred UV or light wavelength ranges (Larsen and Forsyth 2005). Ultimately, both baited and light traps are affected by the placement, habitat, and wind speed surrounding the trap (Barbero et al. 1999, Larsen and Forsyth 2005), which can only select for nearby dung beetles capable of travel and/or flight depending on whether the trap is above- or below-ground. Meanwhile, resource-based extraction avoids competing resource attractiveness, trapping deaths from weather exposure or predators/scavengers, and mobility-based issues—but it still has its own limitations. Flotation is more laborious and less efficient for collecting immobile organisms (e.g., fly puparia—Berkebile et al. 2009) that cannot separate themselves from floating debris quickly. Resource flotation may also still miss groups that arrive, use, and leave the dung quickly (e.g., rollers) or which arrive when most other dung beetles have left (e.g., dry dung arthropods) (Krell 2007), though note that dry dung is not preferred for flotation since it cannot easily break apart and expose beetles to flooded conditions. Regardless of the methodology, a more representative community picture emerges when using a greater sampling area, a longer sampling period, differing methodologies, and collecting during different day lengths and seasons (Davis 2000). Despite the considerations of each collection method, most dung beetle studies employ trapping methodologies for ease-of-use and time-independence, and so extraction-based advantages are overlooked.

Collecting dung beetles for research use, outreach and extension education, or habitat introduction (e.g., Hawaii and Puerto Rico and the United States—Fincher 1986, New Zealand—Dymock 1993, Australia—Doube 2018) is just as important as understanding the benefits of their presence and diversity (Anderson and Loomis 1978). Efficient dung beetle collection methodologies are especially important because of the extreme difficulty of creating artificial dung or building rearing facilities (Blume and Aga 1975), which require large sects of land, animals, money, and specialists – in essence, collecting from existing habitats is easier than recreating habitats in some cases. However, in the case of species introduction, seeding habitats with sterilized organisms reared in a facility is paramount to avoid spreading invasive pests and pathogens within and on dung beetle life stages. This article discusses only trapping methods. Dung beetle trapping methods can destructively sample sensitive (rare, specialist, k-selected) communities and produce unnecessary bycatch, so we focused our efforts on resource-specific flotation and sieving technologies to avoid any ethical issues. Ecologically reducing bycatch helps prevent the unnecessary and potential or real depletion of diverse population of invertebrates and other organisms that keep ecosystems healthy (Read et al. 2006, Spears et al. 2016, Sipolski et al. 2019). Here, we report a time-efficient and cost-effective methodology that collects an abundance of targeted dung beetles with insignificant ill effects.

## Materials and Methods

### Flotation Efficiency and Survival

We assessed the survival and recovery rate of dung beetles using the flotation method across varying times and techniques. Fifty tunnelers (*Onthophagus taurus*) and 200 dwellers (*Labarrus pseudolividus*) were separately seeded into 1,000 g of fresh dung (*n* = 4/treatment) inside containers (18 cm: 25.4 cm—Diameter: Height) lined with 2–3 cm of reused and clean sand for a day. Dung-only controls were floated to record initial dung beetle colonization—none were found. Beetle abundances and mortalities were recorded at 10 s, 30 s, 1 min, 5 min, 10 min, and 30 min of flotation submergence times, with the sieve being removed and the dung swirled to free any remaining dung beetles slightly after 30 min. Recently floated dung beetles were dried and incubated at 10°C to slow activity until mortality could be recorded the next day since this reflects common experimental practices (e.g., collecting and storing organisms the day before their use in an experiment).

### Field Collection

**Fig. 1.**
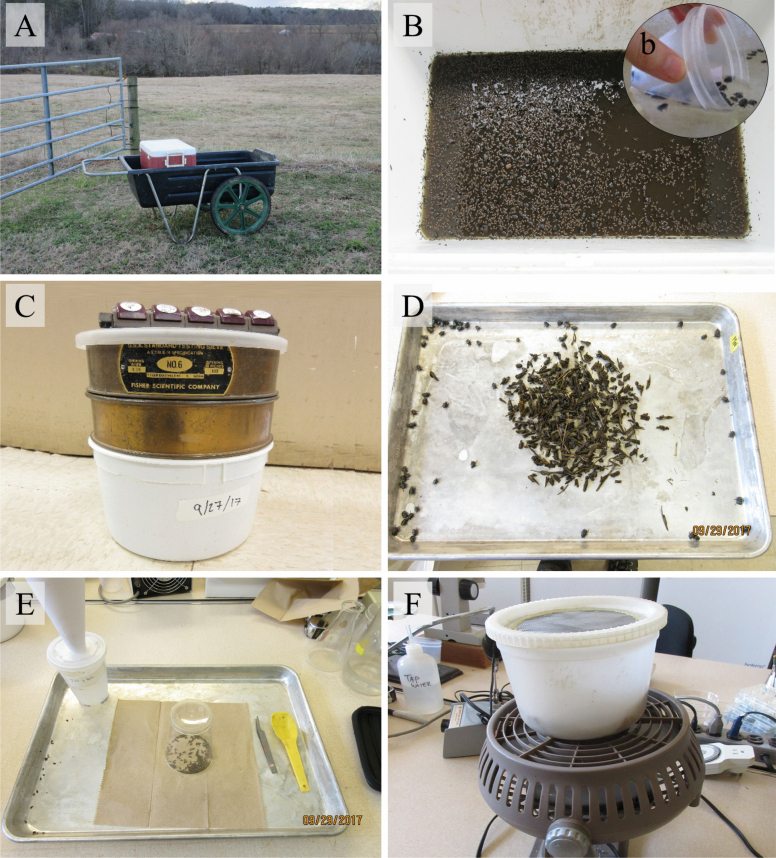
The collection equipment (A), flotation set-up (B) complete with the flow-through cups (b), the self-sieving tower (C), and the sorting of living *O. taurus* (D), and *L. pseudolividus* (E) which are then dried (F).

To test its field practicality and improve its designs, we employed the flotation-sieving technique on heavily colonized dung pats (Fig. 1A) from cattle pastures (NCSU Lake Wheeler Field Lab, Raleigh, North Carolina). Both pictorial (Fig. 1) and textual guides (Table 1) are provided to help researchers’ replicate the design and techniques, but this method will also be reasoned through here. We packed field-colonized dung (no soil, though it can be successfully ‘floated’ as well) loosely underneath one sieve (~1.27 cm—chicken wire can also be used), whose sieve size reflects the length of the largest dung beetle of interest, and which helps keep dung debris submerged during flotation. This dung was flooded with water. In particular, a hose directed water at the side of the flotation bucket to avoid obliterating and dispersing dung particles, but which allowed a gentle stirring of the dung to break it apart. After dung beetles moved through the submerged sieve and floated to the water’s surface (Fig. 1B), they were then scooped up using specimen cups with mesh-bottoms that allowed water (not beetles) to pass through (Fig. 1b). We placed the floated organisms from the specimen cups into a sieving tower topped with a lid to keep them from escaping (Fig. 1C). The ‘sieve tower’ consists of three sieves stacked from largest to smallest sieve size (3.35, 1.50, and 0.85 mm), which allowed dung-based arthropods to self-sort themselves into large, medium, and small-sized organisms based on sieve-size. Dung beetles within the sieve tower were ultimately sprayed with water to 1) keep dung beetles wet to minimize flying; 2) to force the smallest dung beetles to fall through the largest sieves into the smaller sieves; and, 3) to rinse unwanted dung particles through all of the sieves into the waste bucket. Both the beetles actively separately and the spraying of water into the sieve tower forced the mites, sewage water, and other unnecessary particles into the waste bucket. We then placed the living tunnelers onto a tray where they easily separated themselves from each other and the large debris, within minutes, by crawling to the tray edge where they were collected into another container (Fig. 1D). Meanwhile, we separated living dwellers from other small-sized beetles using transparent cups to release unwanted beetles and then funneled them into a container using folded paper (Fig. 1E). This a common form of labor associated with traps that collect multiple species, but in which specific species are targeted, is the releasing unwanted arthropods. We kept these dung beetles in specimen cups with moist towelettes for 24 h in an incubator (10°C) before use during the flotation efficiency and survival experiments.

**Table 1.** A list of suggested items for flotation, self-sorting, and drying arthropods

	Items	Purpose
Collection	Ice cooler + lid	Collect the substrate of interest (dung and/or soil) (Fig. 1A). Mold and cut chicken wire or mesh sieve to your container—the mesh size required depends on the largest arthropod(s) collected.
	Shovel	
	Cart	
	Chicken wire	
Flotation	Specimen cup	Tightly insert the sieve in the container, after placing the dung within, to completely submerge any floatable substrates (Fig. 1B). Gather free-floating arthropods from the water (Fig. 1b) and immediately place them into the sieving tower.
	Window screen	
	Sieving tower	
Self-Sort	Tray	The sieving tower (Fig. 1C) will drain the liquid, separate debris, and self-sort the arthropods based on size. Before capping the tower, spray water through the sieves to: 1) dislodge climbing arthropods and 2) prevent flight.
	Large sieve	
	Medium sieve	
	Small sieve	
	Bucket + lid	
Drying	Bucket	The drying bucket has a mesh lid and bottom to facilitate airflow in one direction via a fan (Fig. 1D). Mass-drying arthropods help during biomass estimation or additional sorting (Fig. 1E and F).
	Window screen	
	Fan	

### Abundance and Biomass Estimation

**Fig. 2.**
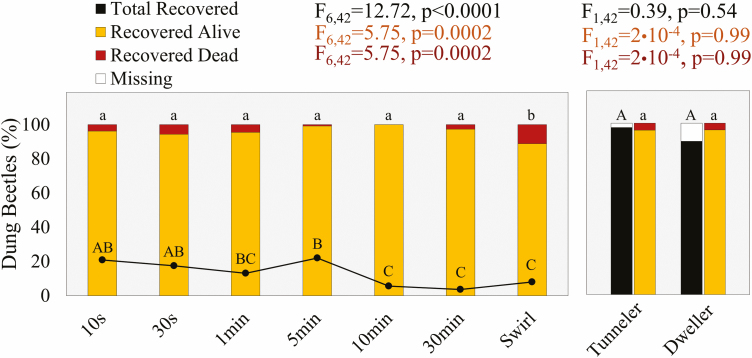
The total dung beetles recovered, living and dead, across either all dung beetle groups (left) or all time periods (right). The main effects ANOVA’s (time or beetle) represent recovery rates per their respective graphs (*F*_df num, df den_ = *F*-value). Differing letters represent differences (*P* < 0.05) for the total dung beetles recovered (uppercase) and the percentage of those recovered that are either living or dead (lowercase).

We assessed dung beetle abundance per biomass to understand beetle capture rates within a practical timeframe. After mass-drying the living dung beetles with a fan (Fig. 1F), we weighed 50 beetles per treatment (*n* = 3) in each experiment (*n* = 5) during 2016–2017 (*n* = 15/treatment) to estimate their abundance and biomass (Fig. 2).

### Statistics

Two-way analysis of variance (ANOVA) and post-hoc Tukey tests were performed on the recovery rates for both dung beetles depending on the total, dead, and alive dung beetle abundances using R (R Development Team, Geneva, Switzerland). The following equations were used for either the statistics or graphs:

$$\begin{array}{l} \begin{array}{ll} & Total\;=\;\left(\#\;captured\;during\;f\;lotation/\#\;inoculated\;in\;dung\right)*100\\ & Alive\;=\;\left(\#\;living\;of\;captured/\#\;captured\;during\;f\;lotation\right)*100\\ & Dead\;=\;\left(\#\;dead\;of\;captured/\#\;captured\;during\;f\;lotation\right)*100\\ & Recovery\;Rates\;=\;rates\;calculated\;at\;time\;X\\ & Cumulative\;Recovery\;=\;the\;sequential\;sum\;of\;\\ & \;recovered\;dung\;beetles\;across\;time \end{array} \end{array}$$

## Results

**Fig. 3.**
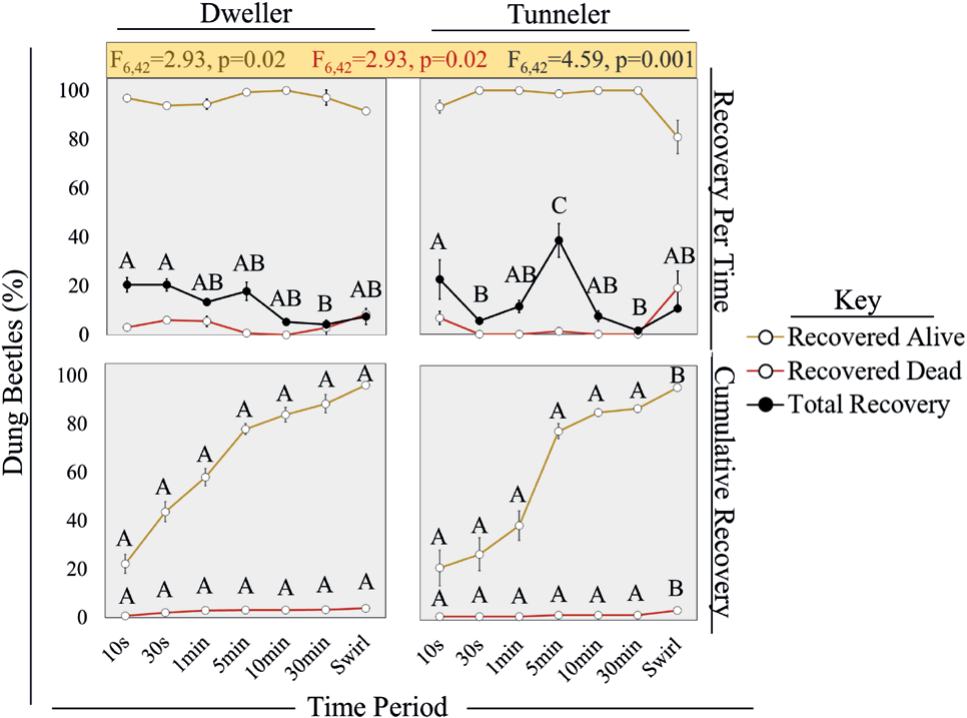
The recovery rate (±SE) of all dung beetles (black) and their respective survival (yellow) and mortality (red) rates per time period (top row) and cumulatively (bottom row) for both *L. pseudolividus* (left column) and *O. taurus* (right column). Interactive ANOVA statistics (beetle:time) shown above graphs (*F*_df num, df den_ = *F*-value) represent the recovery rates per time period for the living, dead, and total dung beetles. Differing letters represent differences (*P* < 0.05) along *x*-axis groups.

We collected 97.5 and 89.4% of *O. taurus* and *L. pseudolividus*, respectively, with >95% survival for both species within the dung pats we seeded. Nevertheless, 2.5% and 10.6% of tunnelers (*n* = 200) and dwellers (*n* = 800) were missing overall, respectively (Fig. 2). Missing dwellers likely escaped during and post-flotation because of their small size and speed compared with the larger and bulkier tunneler. This size difference between beetles may have similarly influenced their collection and mortality rates at specific times. For example, dwellers were steadily collected over a 5 min period; most immediately floated to the water surface once the dung was submerged. Meanwhile, tunnelers were more periodical with 60.5% of all tunnelers collected at the 10 s and 5 min mark specifically (Fig. 3). Regardless, we caught 78 and 72.4% of the total tunnelers and dwellers, respectively, within 5 min of flotation (Fig. 3). Most dead dung beetles (<5% total mortality rate of collected beetles) were found at the beginning and end of the time series (Fig. 2). Nearly 75 and 25% of total dweller deaths, and 40% and 50% of the total tunneler deaths were found within 1 min and on/after 30 min of flotation, respectively (Supp Table 1 [online only]).

**Fig. 4.**
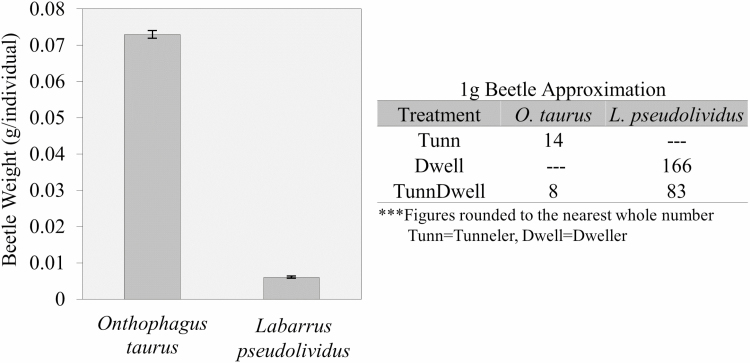
The average mass (±SE) per individual dung beetle (left) and the amount of dung beetles in one gram (right). Used when calculating the total amount collected in the field.

Dung beetle weights (Fig. 4) varied slightly over the season, but the variation was miniscule (<0.001 g per tunneler or 1.45% of bodyweight; <0.0002 g per dweller or 3.92% of bodyweight). In our large-scale flotation methodology field trials (Fig. 1), we often collected >100 g (Tunnelers = 1,394, Dwellers = 16,932) during the height of the dung beetle season within 2 h using only ~16 liters of dung (e.g., four gallon-sized buckets). Time spent on this method depends on how much heavily colonized dung was collected. We saw >98% survival after flotation, but also noted that the activity of dung beetles kept within an incubator at 12°C for 3–5 d was greatly improved if ~5 g dung were added daily.

## Discussion

### To Float or Not to Float?

Flotation is a simple method of collecting and submerging (under water) arthropods from various substrates, including beetles and fly pupae from manure, but not all organisms are collected easily. Flotation is deemed laborious because of the difficulty in collecting the substrate and/or separating organisms from floated debris. So, what arthropod characteristics might lend themselves to easy flotation besides being less dense than water (Miall 1922)? Consider mobility and low oxygen tolerance. Dung beetles, in both temperate (Bertone et al. 2005) and tropical areas (Doube 1991), will move to the soil surface because rainfall loosens soil, softens dry brood balls, and/or creates oxygen-limited (‘hypoxic’) situations compelling escape (Cambefort and Hanski 1991). This means arthropods need to be physically capable of movement through potentially dense materials (soil, water, dung) to escape adverse conditions or harken to advantageous ones. This capability may explain why in our experiment we saw >70% of all dung beetle adult arise within 5 min of flooded dung conditions -suggesting that their natural behaviors increased their capture rates during flotation. Consider also that dung beetles can often tolerate hypoxic conditions because they commonly experience low oxygen (O_2_) concentrations within the dung and soil, including: in saturated soil (e.g., rainfall) or wet dung, within deep burial tunnels, during long brooding times and dense clay soil, and other times when oxygen competes with other gases (carbon dioxide, methane, or nitrogen-containing gases) (Whipple et al. 2013). Low oxygen tolerance (i.e., anaerobic metabolism or discontinuous respiration) may explain why floated *O. taurus* and *L. pseudolividus* adults had survival rates >95% despite being submerged for 30 min. In fact, various dung beetle guilds achieved LT_50_’s between 7 and 37 h (Whipple et al. 2013); thus, checking the LT_50_’s of arthropods of interest may help in selecting oxygen-tolerant organisms and limit any survival concerns.

However, these preferred arthropod characteristics expose some of the potential limitations of this collection methodology. Species, life stages, or groups that are immobile (e.g., brood, eggs, or pupae), insensitive to flooding (i.e., maggots respond more quickly to drying than flooding), and/or prone to drowning during the flotation timeframe should be collected by other means. This method also specifically targets hard-to-reach dung beetles (e.g., dwellers) as opposed to easy-to-filter beetles (e.g., tunnelers, or beetles within the dry soil), which can be more easily extracted in non-water based ways (soil sieving, heat/light extraction), though flotation also works under those conditions. Additionally, the dung type (fine or coarse) and its interaction with water should be considered. The dung we used had some particles sink while others floated, and so the sieves and hose help wash the finest particles into the waste bucket, while the largest particles were caught and easily discarded during sorting. Thus, this method is most efficient and effective when organisms can move quickly to the surface and separate themselves from debris without additional labor, materials, or chemical additives.

Regardless, we observed <5% mortality and attributed the likely causes to incubation conditions, beetle size, and natural death. Interestingly, >90% of all deaths occurred at the beginning (≤1 min) and end (≥30 min) of flotation, which gives us opportunities to improve our floating technique. Consider these contradictions: 1) dung beetles can last for hours submerged in water (Whipple et al. 2013) and so 10–60 s of flotation is unlikely to drown them, but 2) dung beetles need to be alive to dislodge themselves, navigate the sieves, and float to the surface to escape downing. So, what can explain the aggregation of dung beetle deaths? First, we noticed that dung beetle survival and activity in the field, especially for *L. pseudolividus*, was negatively affected by longer incubation periods (>2–3 d) and/or the absence of moisture and dung sources. Here, our dung beetles were without food and water for an entire day before and after flotation prior to mortality counts. This leaves a window of time in which dung beetles may have either died naturally in the dung (see mortality peaks—Fig. 3) and/or during incubation (assumed constant mortality rate across all collection times—Fig. 2). Since many beetles are lighter than water (Miall 1922), the dead dung beetles found <1 min likely died of natural causes and whose light-weight bodies were dislodged by the rush of the water hose, the movement of other living dung beetles, and/or the sway of dung pieces. Meanwhile, dung beetles swirled in the water at 30 min were more likely to have died by drowning unless they were previously dead in the dung, but still floated to the surface because of their light bodyweight (Miall 1922) and they were dislodged from the dung. This may explain why tunnelers had more clumped morality and recovery rates at specific time intervals (10 s, 5 min, 30 min + swirl) as their large size may reduce the chance of dislodgement without effort on part of the dung beetle or researcher (Fig. 3).

### Practicality

Although flotation collection is assumed labor-intensive, time-intensive, and messy (Berkebile et al. 2009), it was an effective method for collecting dung beetles *en masse*. We caught 78 and 72.4% of the dung-occupying tunnelers and dwellers, respectively, within 5 min of flotation; we saw >95% dung beetle survival; and we regularly collected between 50 and 100 g of our targeted dung beetle groups (100 g of Beetles = 1,394 of *O. taurus* or 16,932 of *L. pseudolividus*, which assuming a 50:50 species composition is 95 individuals/g dung. This excludes all other species collected) from field-collected dung (~16 liters) within a few hours. Furthermore, using the flotation-sieving technique, the dung beetles actively separated and sorted themselves from dung debris and other arthropods based on size (Fig. 1) without effort on part of the researcher—a passive technique not described in previous flotation methods. This passive technique, especially using heavily colonized dung, greatly reduced the labor, time, and cost of resource-based extraction methods. Previous flotation methods (Houston et al. 1982, The Northern Tablelands Dung Beetle Express 2005, Krell 2007) used much of the same equipment as us (bucket, sieve or forceps/ladle, stick or hose), which are available to many first- and third-world countries, and so our method is only a revised method of previous methods. Unfortunately, only one of the research articles (Houston et al. 1982) provided some information on the numbers of dung beetles collected, time spent during collection, and volume of dung collected. Houston et al. 1982 used various extraction methods, including flotation, to float anywhere from 80 to 400 liter of dung collecting 10,400–108,600 individuals (130–272 individuals/g dung) within 1–9 h, without species separation or dung collection included in this time count. Therefore, without more literature and data, we suggest that our methodology is equally, if not more, simple, cost-effective (Table 1), and time-efficient when considering our unique data on passive species separation.

### Conclusion

Overall, we show that dung beetle flotation can be time, labor, and cost-effective for biodiversity or collection efforts. We propose that our self-sieving technology can be used for other arthropods, substrates, and methodologies with relative ease.

## Supplementary Material

ieaa083_suppl_Supplementary_Table_1
