# Supplementary material for: Sampling Efficacy and Survival Rates of Labarrus pseudolividus (Coleoptera: Scarabaeidae) and Onthophagus taurus (Coleoptera: Scarabaeidae) Using Flotation and Sieve-Separation Methodology
Source: J Insect Sci. 2020 Nov 2;20(6):18. doi: 10.1093/jisesa/ieaa083 (PMC7751142; doi:10.1093/jisesa/ieaa083)
Supplement: ieaa083_suppl_Supplementary_Table_1 [file ieaa083_suppl_supplementary_table_1.docx]

## SUPPLEMENTARY MATERIALS

| Supplementary Table 1. Dung Beetle Recovery, Survival, and Mortality Rates (%) | | | | | | | |
| --- | --- | --- | --- | --- | --- | --- | --- |
|  |  | Recovered Alive | | Recovered Dead | | Total Recovery | |
|  | n | µ ± se | med | µ ± se | med | µ ± se | med |
| Beetle |  |  |  |  |  |  |  |
| Dwell | 28 | 96.40 ± 0.78 | 97.95a | 3.60 ± 0.78 | 2.05a | 12.77 ± 1.51 | 13.50a |
| Tunn | 28 | 96.38 ± 1.50 | 100a | 3.62 ± 1.50 | 0a | 13.93 ± 2.67 | 9a |
| Time |  |  |  |  |  |  |  |
| 10s | 8 | 95.30 ± 1.51 | 95.16a | 4.70 ± 1.51 | 4.84a | 21.50 ± 4 | 19ab |
| 30s | 8 | 96.88 ± 1.34 | 98.65a | 3.12 ± 1.34 | 1.35a | 13 ± 3.13 | 12.25ab |
| 1m | 8 | 97.32 ± 1.41 | 100a | 2.68 ± 1.41 | 0a | 12.50 ± 1.37 | 12.50bc |
| 5m | 8 | 98.97 ± 0.75 | 100a | 1.03 ± 0.75 | 0a | 28.19 ± 5.30 | 24.25b |
| 10m | 8 | 100 | 100a | 0 | 0a | 6.38 ± 1.27 | 5.50c |
| 30m | 8 | 98.44 ± 1.56 | 100a | 1.56 ± 1.56 | 0a | 2.88 ± 0.78 | 3.25c |
| Swirl | 8 | 87.84 ± 3.93 | 89.68b | 12.16 ± 3.93 | 10.32b | 9 ± 1.69 | 10.25c |
| Beetle:Time |  |  |  |  |  |  |  |
| Dwell:10s | 4 | 96.94 ± 1.32 | 96.57a | 3.06 ± 1.32 | 3.43a | 20.50 ± 2.99 | 19a |
| Dwell:30s | 4 | 93.76 ± 1.40 | 93.43a | 6.24 ± 1.40 | 6.57a | 20.50 ± 2.42 | 19a |
| Dwell:1m | 4 | 94.65 ± 2.13 | 94.47a | 5.35 ± 2.13 | 5.53a | 13.50 ± 1.37 | 13.75ab |
| Dwell:5m | 4 | 99.40 ± 0.60 | 100a | 0.60 ± 0.60 | 0a | 17.88 ± 3.67 | 17.50ab |
| Dwell:10m | 4 | 100 | 100a | 0 | 0a | 5.25 ± 1.31 | 4.50ab |
| Dwell:30m | 4 | 96.88 ± 3.12 | 100a | 3.12 ± 3.12 | 0a | 4.25 ± 0.83 | 4b |
|  |  | Recovered Alive | | Recovered Dead | | Total Recovery | |
|  | n | µ ± se | med | µ ± se | med | µ ± se | med |
| Dwell:Swirl | 4 | 93.17 ± 2.45 | 91.90a | 6.83 ± 2.45 | 8.10a | 7.50 ± 3.30 | 7.50ab |
| Tunn:10s | 4 | 93.66 ± 2.67 | 93.56a | 6.34 ± 2.67 | 6.44a | 22.50 ± 8.06 | 20a |
| Tunn:30s | 4 | 100 | 100a | 0 | 0a | 5.50 ± 1.50 | 6b |
| Tunn:1m | 4 | 100 | 100a | 0 | 0a | 11.50 ± 2.50 | 11ab |
| Tunn:5m | 4 | 98.53 ± 1.47 | 100a | 1.47 ± 1.47 | 0a | 38.50 ± 6.85 | 39c |
| Tunn:10m | 4 | 100 | 100a | 0 | 0a | 7.50 ± 2.22 | 6ab |
| Tunn:30m | 4 | 100 | 100a | 0 | 0a | 1.50 ± 0.96 | 1b |
| Tunn:Swirl | 4 | 82.50 ± 6.85 | 81.67b | 17.50 ± 6.85 | 18.33b | 10.50 ± 0.96 | 11ab |
| [1] Where n=sample size, se=standard error, µ=mean, and med=median  [2] Differing lower case letters represent significant (p<0.05) pairwise comparisons (within  Beetle, between Time) for the means | | | | | | | |
